# Consent Codes: Maintaining Consent in an Ever-expanding Open Science Ecosystem

**DOI:** 10.1007/s12021-022-09577-4

**Published:** 2022-12-15

**Authors:** Stephanie O. M. Dyke, Kathleen Connor, Victoria Nembaware, Nchangwi S. Munung, Kathy Reinold, Giselle Kerry, Mamana Mbiyavanga, Lyndon Zass, Mauricio Moldes, Samir Das, John M. Davis, Jordi Rambla De Argila, J. Dylan Spalding, Alan C. Evans, Nicola Mulder, Jason Karamchandani

**Affiliations:** 1 McGill Centre for Integrative Neuroscience, Department of Neurology & Neurosurgery, Faculty of Medicine and Health Sciences, Montreal Neurological Institute, McGill University, Montreal, QC Canada; 2 Health Level 7 (HL7) & Veterans Health Administration, Washington, D.C, USA; 3 Computational Biology Division, IDM, Faculty of Health Sciences, University of Cape Town, Cape Town, South Africa; 4 Division of Human Genetics, Faculty of Health Sciences, University of Cape Town, Cape Town, South Africa; 5 Broad Institute of MIT and Harvard, Cambridge, MA USA; 6 European Molecular Biology Laboratory, European Bioinformatics Institute, Hinxton, UK; 7 Centre for Genomic Regulation, Barcelona, Spain; 8 Universitat Pompeu Fabra, Barcelona, Spain; 9 Department of Pathology, Faculty of Medicine and Health Sciences, McGill University Health Centre & Montreal Neurological Institute, McGill University, Montreal, QC Canada

**Keywords:** Consent, Open science, Ethics, Data access, Data sharing, Data management

## Abstract

We previously proposed a structure for recording consent-based data use ‘categories’ and ‘requirements’ – Consent Codes – with a view to supporting maximum use and integration of genomic research datasets, and reducing uncertainty about permissible re-use of shared data. Here we discuss clarifications and subsequent updates to the Consent Codes (v4) based on new areas of application (e.g., the neurosciences, biobanking, H3Africa), policy developments (e.g., return of research results), and further practical considerations, including developments in automated approaches to consent management.

## Introduction

As part of the Global Alliance for Genomics and Health (GA4GH) (2016), an international coalition dedicated to increasing the potential of genomic medicine to advance human health through effective and responsible data sharing, a study of the most commonly encountered consent-based data use conditions for the re-use of genomics research datasets generated in both research and clinical settings was undertaken. The study aimed to develop a set of common Consent Codes that would facilitate accurate interpretation of, and respect for, the consent of research participants and patients whose data are shared for Open Science (Dyke et al., 2016). In assigning Consent Codes to datasets, projects sharing data could potentially clearly communicate consent-based data use conditions to data custodians, researchers, and others managing access to data, such as Data Access Committees (DACs), thereby avoiding the need for further expert review of the study’s consent process to determine its permissible uses. Tagging shared research data with Consent Codes was also intended to support automated data discovery and access systems which aim to enable datasets to be either searched for, or accessed, on the basis of researchers’ planned, or approved, purposes of use (e.g., access datasets available for cancer research) (Wong et al., 2017; Cabili et al., 2021). Such automation of data management processes would therefore effectively limit researchers’ use of data according to the Consent Codes assigned to it.

Shortly following their publication and continuously since then, several clarifications and additions were made to the Consent Codes in response to community requirements and new areas of application of the codes, including their use by the Human Heredity and Health in Africa (H3Africa) Consortium (H3Africa Consortium, 2014), in biobanking (e.g., The Neuro C-BIG Repository (Das et al., 2021)) and the pharmaceutical industry, as well as their use in other fields of the life sciences, more specifically, the neurosciences (Tremblay-Mercier et al., 2020). Further insights arose, and clarifications were made, as the Consent Codes were incorporated into informatics tools designed to support automated data access processes (e.g., Health Level 7 International Healthcare Privacy and Security Classification System, GA4GH Data Use Ontology (Lawson et al., 2021), Automatable Discovery and Access Matrix ADA-M (Woolley et al., 2018)).

## Clarifications

### Biological Research

One of the Consent Code primary categories permitting data use for ‘General research use and clinical care’ (GRU(CC)) was initially defined as “For health/medical/biomedical purposes, including the study of population origins or ancestry.” While this code would effectively cover basic biological research that may, ultimately, support beneficial health outcomes, we realized from community feedback, which followed the publication of the codes, that this was not obvious. The description of GRU(CC) was therefore changed to “For health/medical/biomedical purposes *and other biological research*, including the study of population origins or ancestry” in the second version of the Consent Codes (Consent Codes v2, 9 February 2016; see Table 1 for current version of Consent Codes v4, 15 July 2020). While still imperfect as a definition, we were constrained by our effort to harmonize the Consent Codes with pre-existing codes in use by the National Center for Biotechnology Information (NCBI) database of Genotypes and Phenotypes (dbGaP) to classify the hundreds of National Institutes of Health (NIH) research datasets they provide access to (Tryka et al., (2014). The clarification aimed to address the apparent bias towards research with a direct health impact, while maintaining the sense of beneficial research outcomes, even if only as a long-term goal, which is implicit when individuals contribute data to health research. The change also clarified that when permitted using this code, the genetic study of population origins or ancestry may be – at least for the purposes of the research using shared data – unrelated to health.

**Table 1:** Data & Biospecimen Use Categories, Requirements & Permissions (Consent Codes), 15 July 2020 (v4). New codes from Consent Codes v2, v3 and v4 are indicated in bold; modifications are indicated in italics. (Consent Codes v1 was previously published in PLOS Genetics (Dyke et al., 2016))

Consent Codes (v4)		
**Name**	**Code**	**Description**
**Primary Categories** (I^ry^) (pick only one)		
no restrictions	NRES	No restrictions on data use
general research use and clinical care *(as reference data only)*	GRU(CC)	For health/medical/biomedical purposes *and other biological research*, including the study of population origins or ancestry
health/medical/biomedical research and clinical care *(as reference data only)*	HMB(CC)	Use of the data*/biospecimen* is limited to health/medical/biomedical purposes, does not include the study of population origins or ancestry
disease-specific research and clinical care *(as reference data only)*	DS-[XX](CC)	Use of the data*/biospecimen* must be related to [disease]
population origins/ancestry research	POA	Use of the data*/biospecimen* is limited to the study of population origins or ancestry
**Secondary Categories** (II^ry^) (can be one or more extra conditions, in addition to I^ry^ category)		
therapy or drug-specific research	**TDS-[XX]**	Use of the data/biospecimen must be related to [therapy or drug]
other research-specific restrictions	RS-[XX]	Use of the data*/biospecimen* is limited to studies of [research type] (e.g., pediatric research)
no research into specific disease areas	**NDS-[XX]**	Use of data/biospecimen is NOT allowed for research into [certain disease areas]
health-related POA analysis	**HPOA**	Use of data/biospecimen includes analysis of population origins or ancestry ONLY as it relates to health (for HMB and DS datasets)
no “general methods” research	NMDS	Use of the data includes methods development research (e.g., development of software or algorithms) ONLY within the bounds of other data use limitations
research use only	RUO	Use of data is limited to research purposes (e.g., does not include its use in clinical care *as reference data*)
For genetic research datasets/biospecimens:		
genetic studies only	GSO	Use of the data *or biospecimen* is limited to genetic studies only (*e.g.*, no research using only the phenotype *or other health or lifestyle* data)
**Requirements & Permissions** (can be one or more extra conditions, in addition to I^ry^ and II^ry^ categories)		
not-for-profit use only	NPU	Use of the data*/biospecimen* is limited to not-for-profit organizations
non-commercial use only	**NCU**	Use of the data/biospecimen is limited to non-commercial uses
benefit sharing required	**BEN**	Benefits resulting from use of the data/biospecimen (e.g., drug manufacturing) must be shared with the participant community
publication required	PUB	Requestor agrees to make results of studies using the data*/biospecimen* available to the larger scientific community
collaboration required	COL-[XX]	Requestor must agree to collaboration with the [primary study investigator(s)]
return of results (RoR) required	**ROR-[XX]**	Requestor must return individual research results that may be of interest to participants according to the project’s [RoR policy]
return data to database/resource	**RTN**	Requestor must return derived/enriched data to the database/resource
ethics approval required	IRB	Requestor must provide documentation of local IRB/REC approval
geographical restrictions	GS-[XX]	Use of the data*/biospecimen* is limited to within [geographic region]
publication moratorium/embargo	MOR-[XX]	Requestor agrees not to publish results of studies until [date]
time limits on use	TS-[XX]	Use of data*/biospecimen* is approved for [x months]
approval from original study	**OS**	Approval of proposed data/biospecimen use is required from the original study (in addition to data access review)
user-specific restrictions	US	Use of data*/biospecimen* is limited to use by approved users
project-specific restrictions	PS	Use of data*/biospecimen* is limited to use within an approved project
institution-specific restrictions	IS	Use of data*/biospecimen* is limited to use within an approved institution
recontact for questionnaire data	**CQ**	May contact patient/participant to seek consent to provide questionnaire data
recontact for sampling	**CS**	May contact patient/participant to seek consent to provide additional samples
recontact for additional research	**CR**	May contact patient/participant to seek consent to participate in additional research
access to patient medical record	**HR**	The patient/participant has provided consent for access to some data from the patient medical record for research purposes
associated resources available	**ARA-[XX]**	Biospecimens or data from the same individual(s) are available from the following [repository/link]
For biospecimens:		
genetic analysis	**GEN**	May extract DNA, RNA and micro-RNA from biospecimens
cell-lines	**CL**	May produce cell-lines (including stem cells) from biospecimens

### Clinical Care Use & Sharing Healthcare Data for Research

Although we had narrowly defined the “clinical care” uses that the term (CC) encompassed when included in codes such as GRU(CC), this was subsequently clarified in the name and descriptions of all codes with (CC) to help avoid misunderstandings (Consent Codes v3, 23 April 2018). The (CC) permission was included to allow for the use of research data as background information, or what we have called “reference data”, in clinical activities such as diagnostic genetic testing “with the understanding that, due to data quality and the likely consent situation, most of the time, research data would not be used directly in clinical decision-making (by researchers, clinicians, or others) or to provide research participants with their genetic information (Dyke et al., 2016).” A critical point is that an individual’s research data would not be used for their own clinical care without their consent to that use. On the other hand, such research data may be shared and used to improve clinical tools, such as those for variant interpretation, ultimately leading to the contribution of that data to “reference data” used in clinical care. The BRCA Exchange, an open access resource for the clinical significance of BRCA breast cancer gene variants, is a prime example of such use (Cline et al., 2018). Its expert assessment of BRCA variants integrates research findings along with clinical observations (https://brcaexchange.org/). To clarify this permitted type of data use, GRU(CC) is now named ‘general research use and clinical care *(as reference data only)*’ and this has also been added to the ‘Health/medical/biomedical research and clinical care *(as reference data only)*’ HMB(CC) code and ‘Disease-specific research and clinical care *(as reference data only)*’ DS-[XX](CC) code, which is a sub-category of HMB(CC).

The Consent Codes are *research use* codes, designed to facilitate the use of shared research data (also known as secondary use of research data) by clarifying permitted research areas of use as well as other conditions of use. With the recent expansion of precision medicine initiatives, such as the NIH All of Us research program (The “All of Us” Research Program, 2019) and Genomics England 100,000 Genomes Project (Caulfield et al., 2019), the amount of genomic research data that is generated in a clinical environment and coupled with healthcare improvements will only grow. Further, as the promise of genomic medicine is realized in areas such as cancer treatment and prevention, the rationale for one’s research data to be used in their medical care will become stronger. However, with the exception of returning individual research results that could be medically useful to research participants (see "Return of Research Results" section below), the potential use of a participant’s research data for their clinical care does not fall within the current purview of the Consent Codes.

As for contributing healthcare data (e.g., anonymized patient health record data) to research, since their publication, the Consent Codes have been incorporated into the Health Level 7 International (HL7^®^) Healthcare Privacy and Security Classification System (HCS). HL7 is a not-for-profit, ANSI-accredited standards developing organization dedicated to providing a comprehensive framework and related standards for the exchange, integration, sharing, and retrieval of electronic health information that supports clinical practice and the management, delivery and evaluation of health services. The HCS is an international standard document describing the conceptual syntax and semantics suitable for automated labeling and segmentation of protected health care information by access control systems to enforce privacy and security policies (Wong et al., 2017). The HCS specifies how computable access control and provisioning codes can be used to construct “security labels”, which represent the policies for the collection, access, use, and disclosure of governed information and system resources. HCS security labels are metadata assigned to content conveyed in HL7 standards, including HL7 Version 2^®^, HL7 CDA^®^, and HL7 FHIR^®^.

The HCS is based on the approach used by intelligence and national security communities to classify sensitive information in order to restrict access to the “need to know” and its use to “least privilege”. Classification is the level of protection required (the Confidentiality tag) based on the risk of harm that could result from unauthorized access (one or more Sensitivity tags). Based on applicable policy, a security label may also provide sender and receiver instructions as to the context in which the information may be used (Purpose of Use tags, e.g., disease-specific research), any mandated handling caveats (Obligation tags, e.g., de-identify), any prohibited actions (Refrain tags, e.g., no repurposing), as well as any instructions required to be rendered to end users (Privacy Mark tags, e.g., “Confidential”). Additional security label tags include the code and location for the applicable policy (Policy, e.g., Common Rule); restricting access to provisioned members of a group (Compartment, e.g., a specific Research project); and tags used to determine the level of confidence in the authenticity, reliability, and provenance of the information (Provenance, Trust, and Integrity tags) (https://www.hl7.org/implement/standards/product_brief.cfm?product_id=345). While the manner in which security labels are encoded as a set of fields or elements (tags) varies in these different HL7 platform specific syntaxes, the values assigned to tags are all from the same HCS vocabulary, which ensures interoperability and integrity of security label transforms from one HL7 syntax to another, e.g., CDA to FHIR.

HL7 adopted the Consent Codes as Research Purpose of Use codes in July 2017 to achieve compatibility of research use permissions when healthcare data are shared for research purposes, thereby facilitating the pooling of data from research studies and healthcare to conduct further research. Specifically, for non-clinical trial research uses, the HL7 standard now includes codes for biological (BIOHRCH), disease- or discipline-specific (DSHRCH, DISHRCH), population origins and ancestry (POAHRCH), and translational (TRANSHRCH) research uses, all for the purposes of obtaining health care knowledge (see Table 2 for HL7 Research Purpose of Use Codes and their correspondence with Consent Codes; https://www.hl7.org/fhir/v3/PurposeOfUse/vs.html).

**Table 2:** HL7^®^ Healthcare Privacy and Security Classification System (HCS) Research Purpose of Use Codes

Purpose of Use Codes^1^	name	description
HRESCH	healthcare research	To perform one or more operations on information for conducting scientific investigations to obtain health care knowledge. Use of the data includes basic and applied research such as biomedical, population origin or ancestry, translational research, and disease, discipline, specialty specific healthcare research and clinical trial research It excludes clinical trial research, but may be combined with a clinical research purpose of use code to convey that both purposes of use are permitted. May be used in combination with clinical trial purpose of use codes
BIOHRCH (equivalent to the HMB(CC) Consent Code)	biomedical research	To perform one or more operations on information for conducting scientific investigations to obtain health care knowledge. Use of the data must be related to specified biomedical basic or applied research. For example, research on rare plants to determine whether biologic properties may be useful for pharmaceutical development. May be used in combination with clinical trial and other healthcare research purposes of use
CLINTRCH	clinical trial research	To perform one or more operations on information for conducting scientific investigations in accordance with clinical trial protocols to obtain health care knowledge May be post-coordinated or used with other purposes of use such as disease, discipline, specialty, POA, translational healthcare research
CLINTRCHNPC	clinical trial research without patient care	To perform one or more operations on information for conducting scientific investigations in accordance with clinical trial protocols to obtain health care knowledge without provision of patient care. May be post-coordinated or used with other purposes of use such as disease, discipline, specialty, population origins or ancestry, translational healthcare research. For example, a clinical trial conducted on laboratory specimens collected from a specified patient population
CLINTRCHPC	clinical trial research with patient care	To perform one or more operations on information for conducting scientific investigations with patient care in accordance with clinical trial protocols to obtain health care knowledge. May be post-coordinated or used with other purposes of use such as disease, discipline, specialty, population origins or ancestry, translational healthcare research. For example, an "off-label" drug used for cancer therapy administer to a specified patient population
PRECLINTRCH	preclinical trial research	To perform one or more operations on information in preparation for conducting scientific investigation to obtain health care knowledge, such as research on animals or review of patient health records, to determine the feasibility of a clinical trial study; assist with protocol design; or in preparation for institutional review board or ethics committee approval process. May be post-coordinated or used with other purposes of use such as disease, discipline, specialty, population origins or ancestry, translational healthcare research
DSHRCH (equivalent to the DS-[XX](CC) Consent Code)	disease specific healthcare research	To perform one or more operations on information for conducting scientific investigations to obtain health care knowledge. Use of the data must be related to specified conditions, diagnosis, or disease healthcare research. For example, conducting cancer research by testing reaction of tumor cells to certain biologics. May be used in combination with clinical trial and other healthcare research purposes of use
DISHRCH (can be equivalent to RS-[XX], TDS-[XX], or GSO Consent Codes)	discipline specific healthcare research	To perform one or more operations on information for conducting scientific investigations to obtain health care knowledge. Use of the data must be related to specified healthcare discipline. For example, pediatric, aging, genetics, immunology research. May be used in combination with clinical trial and other healthcare research purposes of use
POAHRCH (equivalent to the HPOA Consent Code)	population origins or ancestry healthcare research	To perform one or more operations on information, including genealogical pedigrees, historical records, surveys, family health data, health records, and genetic information, for conducting scientific investigations to obtain health care knowledge. Use of the data must be related to population origins and/or ancestry healthcare research, which may be additionally protected by healthcare specific research confidentiality and privacy policy(s). For example, gathering genetic specimens from a specific population in order to determine the ancestry and population origins of that group. May be used in combination with clinical trial and other healthcare research purposes of use
TRANSHRCH	translational healthcare research	To perform one or more operations on information for conducting scientific investigations to obtain health care knowledge related to evidence based medicine during the course of providing healthcare treatment. Sometimes referred to as "bench to bedside", which is the iterative feedback loop between healthcare research and clinical trials with input from information collected in the course of routine provision of healthcare. For example, by extending a patient encounter to conduct a survey related to a research topic such as attitudes about use of a wellness device that a patient agreed to use. May be used in combination with clinical trial and other healthcare research purposes of use

^1^https://www.hl7.org/fhir/v3/PurposeOfUse/vs.html

Security Labels for Research, which included Research Purpose of Use codes, have been demonstrated by the US Office of the National Coordinator (ONC) during the ONC Patient Choice project at Healthcare Information and Management Systems Society (2017) and by the Research Patient Choice pilot run by REACHnet, a member of the National Patient-Centered Clinical Research Network (PCORnet) (Bradford and Carton). Both demonstrations focused on how security labels that encode a study subject’s choices are captured in a FHIR Consent Directive using FHIR Consent Forms, and are then matched with a researcher’s access request to determine what information the researcher was permitted to access.

## New Codes

### “Sensitive” Research & “Vulnerable” Populations

#### The Experience of the Human Heredity and Health in Africa (H3Africa) Consortium

The Human Heredity and Health in Africa (H3Africa) Consortium, involving African researchers in 30 countries across the continent, is generating genotype and phenotype data from a variety of African populations to study the genetic and environmental factors underlying diseases of relevance to the continent (Mulder et al., 2018). In order to implement equitable and responsible sharing of the data, the consortium has developed a data sharing, access and release policy that promotes the public release of the data *via* a controlled access procedure, while providing sufficient time for African researchers (who may have limited resources compared to developed countries) to analyze their data, and protecting the rights of the participants and the consents given (Yakubu et al., 2018). Data sharing is particularly sensitive due to previous exploitation of samples from the continent, so it is essential to build trust in the data custodians and ensure that secondary use is in line with the original consents (Editorial, 2019).

During a workshop, H3Africa data managers helped to map consent-based data use permissions and restrictions for each project to the Consent Codes. In adopting the Consent Codes to implement H3Africa’s data sharing plans, we realized that certain areas of research may be considered particularly sensitive by research participants, and if permitted for secondary uses of data, might discourage research participation. For example, in the African context, many research participants may wish to exclude research into HIV and AIDS from secondary research uses of data and samples for fear of stigmatization. As such areas of sensitivity could well be a subset of a broader permissible research area (e.g., HIV and infectious disease), this situation called for an extra “exclusion” code, as opposed to the “permission” codes for disease-related research DS-[XX] (Consent Codes v3, 23 April 2018, ‘No research into specific disease areas’ NDS-[XX] code).

Further, in some H3Africa projects, as an extra measure to protect the interests of research participants, proposed secondary uses of data and biospecimens must be approved by the research ethics board that approved the original study in addition to standard access review by the H3Africa Data and Biospecimen Access Committee. This is distinct from requirements for local research ethics review of the study using shared resources (‘Ethics review required’ IRB code). Therefore, a new code was added to reflect this requirement (Consent Codes v4, 15 July 2020, ‘Approval from original study’ OS code).

Finally, benefit sharing – the sharing of advantages and profits derived from research with the communities that are participating in the research — may be required for access to some of the H3Africa data and biospecimens (CIOMS, 2016) (Schroeder, 2007). A benefit sharing code was therefore added to the list of Consent Code Requirements (Consent Codes v4, 15 July 2020, ‘Benefit sharing required’ BEN code). H3Africa also required a ‘non-commercial use only’ requirement code (Consent Codes v4, 15 July 2020, NCU code) based on some local communities’ agreements. This code is broader than the ‘not-for-profit use only’ NPU code, which could allow not-for-profit commercial uses. The Consortium has also developed specific permission codes for various types of commercial development based on shared resources to further specify acceptable commercial uses, namely for drug development, diagnostic test development, and a broad category for ‘other commercial use’. Under current genomic data access models and Open Science policy, fine-grained restrictions on specific types of commercial development based on the research using shared resources, as well as other related conditions of use (e.g., patenting and licensing terms), are typically specified along with other conditions of data use in the Material Transfer or Data Access Agreements that must be signed prior to data access. However, clearly communicating such conditions upfront for potential applicants and enabling dataset selection on that basis presents advantages. Further, this approach might be suitable in the context of the new and emerging Registered Access data access policy model (Dyke et al., 2016, 2018), which uses a general set of terms of access, that include respecting any dataset-specific restrictions, rather than case-by-case Data Access Agreements. We therefore expect this approach will be helpful and could be extended to standard categories that would apply across the life sciences.

#### Ethnicity Data in Health Research

**Fig. 1:**
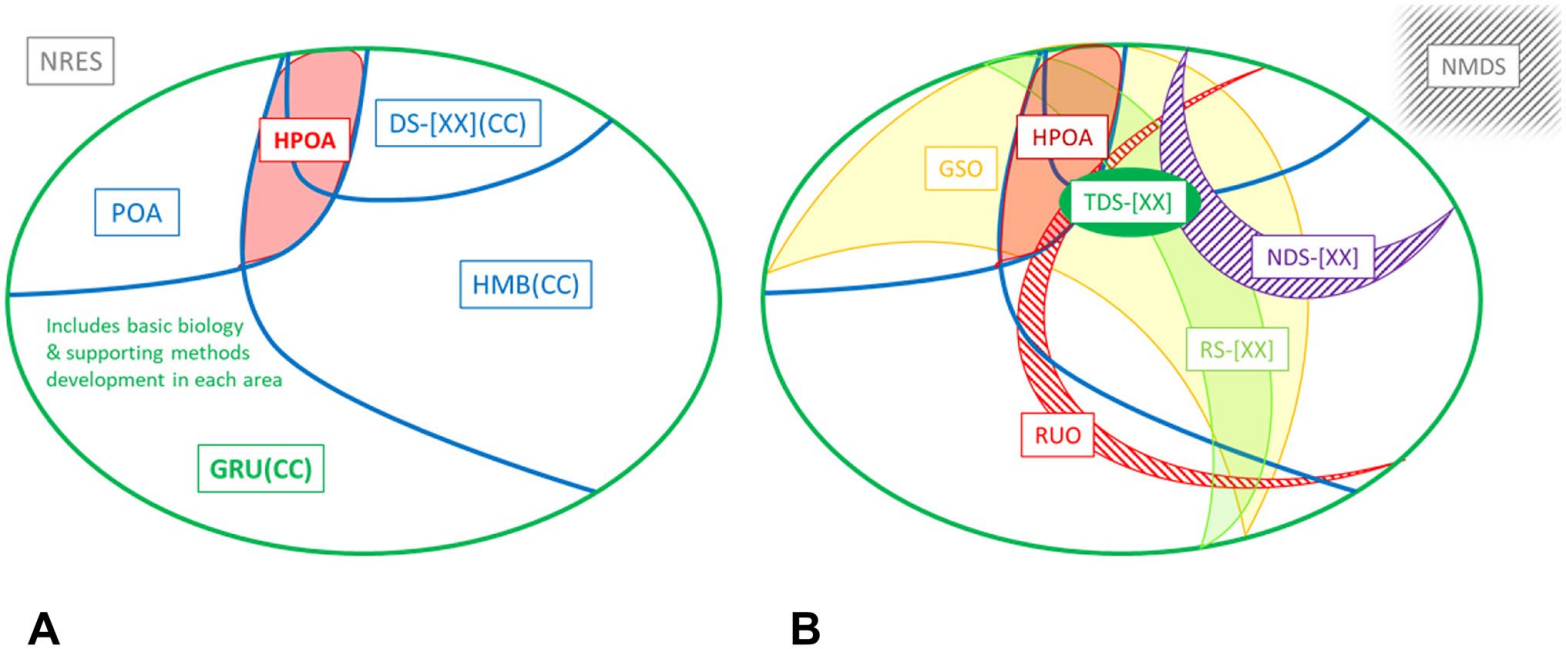
Map of Consent Code Primary (I^ry^) and Secondary (II^ry^) Codes. **A**. I^ry^ Consent Codes (NRES, GRU(CC), HMB(CC), DS-[XX](CC), POA). Shows where the ‘health-related POA analysis’ (HPOA) II^ry^ Consent Code falls. NRES indicates that there are no restrictions at all on data use (“Open Access” code). **B**. II^ry^ Consent Codes (TDS-[XX], RS-[XX], NDS-[XX], HPOA, NMDS, RUO). II^ry^ codes are “modifiers” of the I^ry^ Consent Code selected. Hashed surfaces show exclusions to permitted uses: 1) certain disease-related research for NDS-[XX]; 2) the use of data as reference data in clinical care (CC) for RUO; and 3) general methods development research beyond that which directly supports permitted research for NMDS

In considering both the evolution of genomics research and the application of Consent Codes to other areas of the life sciences that might not involve genetic data, an emerging challenge concerned the use of ethnicity data in health research. There is growing understanding and awareness that population-based analysis across a diversity of populations is critical to assess genetic risk (Martin et al., 2017; Manrai et al., 2016; Chi et al., 2019; Swart et al., 2020; Gurdasani et al., 2019). Distinct consent to in-depth study of an individual’s genome for population origins and ancestry research (POA code, either alone or along with health research as part of GRU(CC)) has been essential in genomics given that such research raises concerns for many individuals as well as communities, such as indigenous populations. In health research contexts, however, including genetic medicine, the inclusion of ethnicity data for analysis is narrower in scope than the range of research permitted by the POA code. For many studies, the inclusion of voluntarily-provided, self-reported ethnicity information would be considered part of health-related research consent permissions (HMB(CC) or DS-[XX](CC)), but its use to study broader population origins and ancestry questions would not. Having said this, health-related analysis by ethnicity may still be considered sensitive and potentially stigmatizing by some research participants and groups (Santos, 2008). An explicit ‘health-related POA analysis’ code would therefore allow either opt-in or opt-out options for such use of population origins or ancestry data (as it could be derived from genetic data), and to specify whether such analysis is permitted for a particular study dataset (see Fig. 1A; Consent Codes v4, 15 July 2020, HPOA). While the addition of this new secondary Consent Code could raise questions about the use of existing datasets (e.g., an HMB(CC) dataset which includes ethnicity data), we anticipate that data providers (as well as data users via the providers) will be able to seek clarity of permissions based on their knowledge of the original studies and communities involved in research. The HPOA code may benefit participants from communities that would otherwise be averse to population origins and ancestry research by providing for this narrower health-related focus. In general, people are more willing to contribute data for health research compared with other research purposes. Furthermore, it is likely to be helpful to the research community and to the potential benefits that participants may derive from research in emerging contexts which bring together existing datasets (such as an individual’s research and healthcare data), or when datasets are collected and stored for very broad use, such as in biobanking, both of which augment the challenges for participants to fully appraise the use of their data. The HPOA code corresponds to the POAHRCH HL7 Research Purpose of Use code.

### Biobanking

Following the initial publication of the Consent Codes, we received a request from the biobanking community to include a ‘Requirement’ code for one of the common conditions of their use, which is that users of biobank materials contribute any data generated from the materials (e.g., genetic sequencing data) to further expand the biobank’s collection and benefit future researchers accessing these resources (Consent Codes v2, 9 Feb 2016, ‘Return data to database/resource’ RTN code).

The Montreal Neurological Institute and Hospital (The Neuro) C-BIG Repository is an Open Science collection of biological samples, clinical information, brain imaging, and genetic data from patients with neurological disease as well as from healthy control subjects (Das et al. 2021). This biobank was created as part of The Neuro’s Tanenbaum Open Science Institute to empower researchers to develop insights into the biology underlying neurological disease and facilitate the development of new treatments for patients with neurological diseases (Poupon et al., 2017). In implementing Consent Codes for C-BIG’s Open Science plans, which uses the Longitudinal Online Research and Imaging System (LORIS) research data management platform (Das et al., 2016, 2017), we came across further “biobanking” consent-based permissions that could potentially be useful to communicate to researchers seeking to access shared data and biospecimens, and which may apply to projects other than biobanks. The first of these were permissions to (re)contact research participants and patients that had participated in the biobank, either to seek their consent to provide additional questionnaire data or biospecimens to be used within the scope of the research that they had previously consented to (Consent Codes v3, 23 April 2018, ‘Recontact for questionnaire data’ CQ code and ‘Recontact for sampling’ CS code), or to invite them to participate in additional areas of research and new research projects (Consent Codes v3, 23 April 2018, ‘Recontact for additional research’ CR code). In addition, one other Consent Code was added to indicate that permission had been granted for access to some of the data from the patient medical record for research purposes (Consent Codes v3, 23 April 2018, ‘Access to patient medical record’ HR code).

These codes derive from the longer-term and often more continuous consent relationship that is typical of biobanking projects, though not specific to them. In the current research environment, these “biobanking” codes would simply signal to users that there may be an opportunity to expand the research dataset or recruit research participants through collaboration with the teams contributing to Open Science, be it a biobank or another Open Science resource. There is growing interest in identifying potential candidates for research, especially for recruitment in clinical trials, based on their molecular profile, e.g., particular genetic variants. Shared data from willing research participants could facilitate this type of recruitment.

Two more biobanking ‘Permission’ codes for specific uses of biospecimens were included based on the experience of C-BIG: the first to specify the participant’s consent to the generation of cell-lines (including stem cells) from their biospecimens (Consent Codes v4, 15 July 2020, ‘Cell-lines’ CL code); the second for consent to the extraction of DNA, RNA and micro-RNA for genetic analysis of biospecimens (Consent Codes v4, 15 July 2020, ‘Genetic analysis’ GEN code).

Finally, in the H3Africa context, as the biobanking (biospecimen) and data repositories were separate and distributed, there was also a need to expand the codes to flag the availability of biospecimens (from the same research participants whose data are available) in one of the H3Africa regional biorepositories, so that researchers who have a need to perform further laboratory experiments are alerted to their availability. Along with suitable research participant ID systems, this new code will help link biobank and data resources (Consent Codes v4, 15 July 2020, ‘Associated resources available’ ARA-[XX] code).

### Clinical Trials

Following United Nations recommendations that governments require the sharing of all clinical trial data, the European Clinical Trial Regulation coming into force, and policy proposals by medical journal editors to make clinical trials data available at the time of publication, the sharing of clinical trials data for further research purposes has rapidly expanded over the past few years (United Nations, 2016; Taichman et al., 2016). As it was for genomics and neuroscience data, clinical trial data sharing has been promoted and facilitated by the use of widely recognized metadata standards (Ohmann et al., 2017). However, in collaborating with teams developing data sharing IT resources with the pharmaceutical industry, we encountered a common limitation arising in this context for the sharing of clinical trials data. Standardly-used consent materials would typically require that secondary use of data be related to the therapy or drug that was the focus of the clinical trial from which data originate (Consent Codes v3, 23 April 2018, ‘Therapy or drug-specific research’ TDS-[XX] code). This restriction is a secondary code and may also be within a disease-specific area (DS-[XX](CC)), as well as in addition to an ‘other research-specific restrictions’ code (RS-[XX] code), e.g., permitting pediatric research only.

Additionally for clinical trials, the HL7 Research Purpose of Use codes for healthcare data presented above include codes for the use of data for pre-clinical trial (PRECLINTRCH) and clinical trial health research that is either linked to the provision of patient care or not (CLINTRCHPC; CLINTRCHNPC).

### Return of Research Results

Expectations concerning the return of individual research findings to research participants are rapidly evolving, particularly in genomics (Jarvik et al., 2014; Knoppers et al., 2016; Dyke et al., 2019; Vears et al., 2021), largely due to progress in clinical interpretation of genetic variants (ACMG Board of Directors, 2015). Managing more extensive return of results policies can become rather complex in the context of Open Science, particularly for biobanking resources, which may involve not only extensive genetic data, but often also imaging data as well as biospecimens for further analysis (Wolf et al., 2012). Indeed, in these situations, incidental and other relevant findings that must be returned to participants according to a specific return of results policy might eventually be found by a research team without much connection to the original study team or biobank. Although we foresaw greater emphasis on the return of results emerging at the time of first publication of the Consent Codes, given the challenges, we initially suggested using the ‘collaboration required’ code (COL-[XX] code) for projects with return of results requirements. Return of results requirements could therefore be specified either in collaborative agreements or Data Access and Material Transfer Agreements. However, as both return of results and Open Science policy continue to evolve, (Lewis, 2021) and with the Registered Access data access model designed to provide broad access to data without tailored Data Access Agreements, we have now added a new code for the requirement to return individual research results for biobanks and Open Science projects that aim to enable the report of potentially useful individual findings (Consent Codes v4, 15 July 2020, ‘Return of results required’ ROR-[XX] code). This code will serve to alert potential applicants and users to requirements in this regard, and it will need to be accompanied by details of the specifics of, and procedures for, individual return of results policies (e.g., which results must be returned).

## Conclusion/Future Developments

### Beyond Genomics

Consent Codes were originally developed based on an analysis of the most common consent-based data use permissions and restrictions that existed for the hundreds of genetic research datasets shared through the major public genome repositories (dbGaP in the USA and the European Genome-phenome Archive (EGA) in Europe (Lappalainen et al., 2015)). They therefore allow for representation of restrictions arising from a large pool of existing consent agreements, some of which were written years ago, in order to leverage the value of a large corpus of existing data. In adopting the codes for broader purposes, such as biobanking, and their use in other fields, such as the neurosciences, they have also proven well suited to other areas of the biomedical and life sciences, as well as to indicate restrictions on the use of biospecimens along with data. There was only one code that could only apply to genetic research datasets or biospecimens and it is now labelled as such in the code table (‘Genetic studies only’ GSO code), and there are now two new codes that apply only to biospecimens. Where relevant, Consent Code descriptions have been updated to specify their potential application to both data and biospecimens (Consent Codes v4, 15 July 2020). Consent Codes now also include codes that can be used to capture restrictions and requirements arising from increased awareness and sensitivity around vulnerable populations. As we continue to collaborate with a wide range of Open Science initiatives, we expect further Consent Code additions to reflect the needs and concerns of communities participating in research.

There can sometimes be tensions between the concept of shared and open data and the need to preserve research participants’ privacy interests and protect vulnerable populations. We nevertheless aim to encourage sharing of more data through the use of clear and widely compatible restrictions, and through clearly specifying these permissions, restrictions, and obligations, to help to extend biomedical research to new frontiers.

Big data analytics is sweeping across all areas of health research and increasingly relies on access to clinical data with clear use permissions to rapidly advance knowledge and healthcare practices. In collaborating with HL7 to align its Research Purpose of Use codes with Consent Codes, our aim was to broaden access to healthcare data that patients are willing to make available for research purposes and facilitate its pooling with datasets from research projects.

### Automated Data Access Systems

Data access policy is undergoing transformations both to adapt to the current scale of data sharing across the life sciences and to facilitate novel analyses, such as machine learning, that often depend on bringing together several existing datasets (Dyke, 2020). Considerable efforts are underway to enable greater automation of data discovery and access processes (The Beacon Project (Fiume et al., 2019); The Matchmaker Exchange (Philippakis et al., 2015); GA4GH Passport (Voisin et al., 2021); DUOS Data Use Oversight System (Cabili et al., 2018; 2021)), including several informatics tools to support the identification and selection of datasets based on data use permissions such as Consent Codes (GA4GH Data Use Ontology (DUO) ( Lawson et al., 2021); Automatable Discovery and Access Matrix (ADA-M)) (Woolley et al., 2018). A Consent Code to DUO term correspondence table is provided in Table 3. There is also ongoing work on computable research consents at HL7, in particular using FHIR.

**Table 3:** Consent Codes v4 to GA4GH DUO (version 2021–02-23) term correspondence table

**Consent Code**	**DUO term**
**I**^**ry**^** Codes**	
NRES	DUO:0000004
GRU(CC)	DUO:0000005 (Attn: not to be confused with the much broader GRU DUO term DUO:0000042)
HMB(CC)	DUO:0000006
DS-[XX](CC)	DUO:0000007
POA	DUO:0000011
**II**^**ry**^** Codes**	
TDS-[XX]	not in DUO
RS-[XX]	DUO:0000012
NDS-[XX]	not in DUO
HPOA	not in DUO
NMDS	not in DUO (Attn: The DUO NMDS term DUO:0000015 prohibits any methods development research)
RUO	DUO:0000014
GSO	DUO:0000016
**Other Requirement & Permission Codes**	
NPU	DUO:0000045
NCU	not in DUO (Attn: The DUO NCU term DUO: 0000046 defines non-commercial use as data can be used by commercial organisations for research purposes, but not commercial purposes.)
BEN	not in DUO
PUB	DUO:0000019
COL-[XX]	DUO:0000020
ROR-[XX]	not in DUO
RTN	DUO:0000029
IRB	DUO:0000021
GS-[XX]	DUO:0000022
MOR-[XX]	DUO:0000024
TS-[XX]	DUO:0000025
OS	not in DUO
US	DUO:0000026
PS	DUO:0000027
IS	DUO:0000028
CQ	not in DUO
CS	not in DUO
CR	not in DUO
HR	not in DUO
ARA-[XX]	not in DUO
GEN	not in DUO
CL	not in DUO

Through GA4GH, we work with these informatics tools to try and maintain compatibility where possible. The EGA, for example, has adopted the use of Consent Codes through DUO for a majority of its managed access datasets. In addition, its policy schema metadata was updated so that all data submissions are now tagged and the codes displayed with datasets, which enables search and filtering by consent and data use permissions.

Efforts to further articulate research use cases for the development of the specific research-related security labeling codes within HL7 are also under consideration. The benefits of computably enforcing Consent Code Requirements and Permissions, for example, are of paramount importance to furthering interests in health and life sciences goals, particularly given the emergence of artificial intelligence as a research tool that has yet to be fully enabled in contexts with complex ethical-legal constraints.

The expansion of the Open Science ecosystem across different fields of research and between healthcare and research settings is very promising for future research prospects. Standardizing and automating the retrieval of data access and use conditions offers great opportunities to both reduce the costs and administrative burden of data management, and speed up data access procedures for researchers. There are also clear benefits to having more data shared with fewer restrictions. However, resolving and clarifying even slight nuances in summary descriptions of consent-based data use permissions and requirements will be essential in order for automated systems to support the responsible management of data access and use according to accurate and reliable descriptions of research participants’ understanding of data sharing plans.
